# Cytokine and Chemokine Levels in Patients with Severe Fever with Thrombocytopenia Syndrome Virus

**DOI:** 10.1371/journal.pone.0041365

**Published:** 2012-07-24

**Authors:** Baocheng Deng, Shujun Zhang, Yingzhi Geng, Yuzhong Zhang, Yuncheng Wang, Wenqing Yao, Ying Wen, Wei Cui, Ying Zhou, Qiuhong Gu, Wen Wang, Yu Wang, Zhen Shao, Yanli Wang, Chengbo Li, Donglei Wang, Yitong Zhao, Pei Liu

**Affiliations:** 1 Department of Infectious Diseases, The First Affiliated Hospital, China Medical University, Shenyang, Liaoning Province, China; 2 Department of Infectious Diseases, Kuandian Country Hospital, Dandong, Liaoning Province, China; 3 Liaoning Province Center for Disease Control and Prevention, Shenyang, Liaoning Province, China; German Primate Center, Germany

## Abstract

**Background:**

Severe fever with thrombocytopenia syndrome virus (SFTSV), which can cause hemorrhagic fever–like illness, is a newly discovered bunyavirus in China. The pathogenesis of SFTSV infection is poorly understood. However, it has been suggested that immune mechanisms, including cytokines and chemokines, play an important role in disease pathogenesis. In the present study, we investigated host cytokine and chemokine profiles in serum samples of patients with SFTSV infection from Northeast China and explored a possible correlation between cytokine levels and disease severity.

**Methods and Principal Findings:**

Acute phase serum samples from 40 patients, diagnosed with SFTSV infection were included. Patients were divided into two groups – severe or non-severe – based on disease severity. Levels of tumor necrosis factor (TNF)-α, transforming growth factor (TGF)-β, interleukin-6, interferon (IFN)-γ, IFN- γ-induced protein (IP)-10 and RANTES were measured in the serum samples with commercial ELISAs. Statistical analysis showed that increases in TNF-α, IP-10 and IFN-γ were associated with disease severity.

**Conclusions:**

We suggest that a cytokine-mediated inflammatory response, characterized by cytokine and chemokine production imbalance, might be in part responsible for the disease progression of patients with SFTSV infection.

## Introduction

Severe fever with thrombocytopenia syndrome (SFTS) is a tick-borne hemorrhagic fever–like illnesses caused by severe fever with thrombocytopenia syndrome virus (SFTSV), which is a member of the Bunyaviridae family and newly identified in Central and Northeast China [1]. SFTSV infection characterized by fever, thrombocytopenia, leukocytopenia, and multiorgan dysfunction produces a broad spectrum of clinical manifestations, which ranges from an acute self-limited febrile illness to various grades of a severe disease with a reported case–fatality rate varying between 12% and 30% [2]. Humans become infected through tick bites, contact with blood from SFTS patients, and personal contact [3]. The pathogenic mechanism in patients with SFTSV infection is at least partly immune-mediated, which may play an important role in determining the severity and clinical outcome [4]. Other viruses, such as Hantaviruses and Crimean–Congo hemorrhagic fever virus, cause cytokine activation, and an uncontrolled release of cytokines has been observed in filovirus infection similar to that seen in sepsis caused by Gram-negative bacteria [5]. Until now, the pathogenesis of SFTSV infection has not been clearly defined, and cytokine and chemokine studies with respect to SFTSV infection are lacking. The present study determined the concentrations of tumor necrosis factor (TNF)-α, transforming growth factor (TGF)-β, interleukin (IL)-6, interferon (IFN)-γ, IFN-γ-induced protein (IP)-10 and RANTES in serum samples of SFTS patients during the 2011 endemic in Northeast China, and correlated them with disease severity. We also analyzed T-cell subgroups and their possible role in disease severity.

## Materials and Methods

### Ethics Statement

Patients all gave written consent to the participation in our study. Permission to perform this study was given by the Ethics Committee of China Medical University.

### Patients and Clinical Samples

Fifty-seven patients with suspected SFTSV infection treated in our hospitals between April and November 2011 were tested for SFTSV at admission. A suspected case was defined as one with acute fever, in which the pathogen could not be identified, and at least one of the following laboratory tests: thrombocytopenia, leukocytopenia or a history of arthropod bites. Confirmed cases were defined by a positive result in a quantitative RT-PCR or a positive result for IgM antibody to SFTSV. Testing was also performed for detection of human granulocytic anaplasmosis, HFRS, Crimean–Congo hemorrhagic fever, *Leptospira*, *Salmonella*, *Rickettsia* or *Brucella*; and EBV, cytomegalovirus, hepatitis A, B, C and E virus were also investigated.

Plasma samples were obtained from patients during the acute phase of their illness. After sampling, serum was extracted and immediately frozen at –80°C until serum analysis. The clinical course and laboratory data of the patients were recorded prospectively on individual forms. Severe SFTSV infection was defined as any person who required admission to an intensive care unit and met at least one of the following criteria: ARDS, heart failure, liver failure, shock or disseminated intravascular coagulation. Data on demographic characteristics and laboratory measures were expressed as mean ±SD or median.

In addition, serum samples from 40 healthy volunteers (who did not have a febrile illness in the preceding week and were not epidemiologically linked to the endemic) were also included as controls.

### Measurement of Plasma Cytokines

Blood samples coated with EDTA were collected from patients on admission during the acute phase of SFTSV infection. Plasma was separated by centrifugation (3000 *g* for 10 min, 4°C) and stored at –80°C until analyzed. Serum levels of TNF-α, TGF-β, IL-6, IP-10, IFN-γ and RANTES were measured retrospectively by an ABC ELISA kit (Research & Diagnostics, Minneapolis, MN, USA), in accordance with the manufacturer’s instructions.

### Statistical Analysis

Means for continuous variables were compared using independent-group Student’s *t* tests when the data were normally distributed; otherwise, the Mann-Whitney test was used. Proportions for categorical variables were compared using the χ^2^ test, although Fisher’s exact test was used when the data were sparse. Correlation was assessed using Pearson’s test. Significance was set at *p*<0.05, using two-sided comparisons. Results were analyzed using SPSS for Windows version 17.0 (SPSS, Chicago, IL, USA).

## Results

Forty patients, comprising 32 SFTS cases confirmed by RT-PCR and eight by ELISA were admitted from hilly areas of Northeastern China; six (15.0%) of whom died. The mean age of the patients was 55.7±14.7 years (range, 18–89 years), and 11 (27.5%) were female (Table 1). Fever, fatigue, anorexia, diarrhea, nausea and myalgia were the most frequent symptoms in all patients. The major clinical symptoms in critical cases were disturbance of consciousness, arrhythmias, pancreatitis, serious pneumonia, capillary leakage, hypotension and shock, in addition to fever, thrombocytopenia, and leukopenia. Hemorrhagic-fever-like symptoms were also observed in the patients. All the patients suffered fever during the course of the disease. The median interval between the onset of illness and the day in which serum samples were obtained at admission was 5.5 days (range, 1–10 days). The mean length of hospital stay was 9.7±5.2 days (range, 1–22 days). Six (15.0%) of 40 patients died of acute left ventricular failure, multiorgan dysfunction or aplastic anemia within 4–18 days of admission, while the remaining 34 patients (85.0%) survived. Nine (22.5%) of 40 patients met the criteria for severe disease. Pleural and pericardial effusions were observed in eight (20.0%) and three (7.5%) patients, respectively.

**Table 1:** Characteristics of 40 SFTSV-infected patients included in the study.

Characteristic	Value
**Female sex – no./total no. (%)**	11/40 (27.5%)
**Age – yr**	
** Mean**	55.7±14.7
** Range**	18–89
**History of confirmed tick bite – no./total no. (%)**	4/40 (10%)
**Clinical outcomes**	
** Incubation period – d**	
** Mean**	15.0±7.1
** Range**	5–20
**Duration of fever – d**	
** Mean**	6.7±2.5
** Range**	1–12
**Length of hospital stay – d**	
** Mean**	9.7±5.2
** Range**	1–22
**Interval between onset and admission – d**	
** Median**	5.5
** Range**	1–10
**Death - no./total no. (%)**	6/40 (15.0%)

There was a significant difference in the interval between onset and admission between severe and non-severe cases (median, 7.0 vs 5.0 days, respectively; *p* = 0.016) (Table 2); however, age, sex ratio, duration of fever, and length of hospital stay were similar between the severe cases with SFTSV infection and those with non-severe infection (*p*>0.05). Apart from the patient who died from aplastic anemia, who had coexisting hypertension and coronary heart disease, the other patients with severe disease had no evidence of pre-existing comorbidity.

**Table 2:** Differences in clinical and laboratory characteristics between severe and non-severe cases of SFTSV infection.

	Severe cases (n = 9)	Non-severe cases (n = 31)	All patients (n = 40)	*P* value^a^
	Median (range)	Median (range)	Median (range)	
**Days between onset and admission**	7.0 (1–10)	5.0 (3–10)	5.5 (1–10)	0.016
**CRP, mg/liter**	13.0 (3.7–64.5)	5.0 (0.2–79.1)	5.5 (0.2–79.1)	0.019
**CK, U/liter**	1600.0 (54.0–23,000.0)	462.5 (35.0–3847.0)	478.1 (35–23,000.0)	0.029
**LDH, U/liter**	2342.0 (667.2–8475.0)	494.6 (146.5–3304.1)	585.9 (146.5–8475.0)	0.033
**Platelet count, per mm^3^**	24,000(16,000–40,000)	59,000 (10,000–91,000)	43,500 (10,000–91,000)	<0.001
**Calcium, mmol/liter**	1.75 (1.4–1.9)	1.98 (1.6–2.3)	1.87 (1.4–2.3)	0.002
**Albumin levels, g/liter**	27.0 (22.8–28.7)	32.8 (23.3–44.1)	30.1 (22.8–44.1)	<0.001

Data are median (interquartile range).

a Severe versus non-severe.

All of the patients had thrombocytopenia, and elevated aspartate aminotransferase (AST), alanine aminotransferase (ALT) and lactate dehydrogenase (LDH) levels. Of the clinical laboratory parameters tested, C-reactive protein (CRP), creatinine phosphokinase (CK) and LDH levels were significantly higher; platelet count, calcium and albumin levels were significantly lower in the patients with severe SFTSV infection, compared with those with non-severe infection (Table 2).

**Figure 1:**
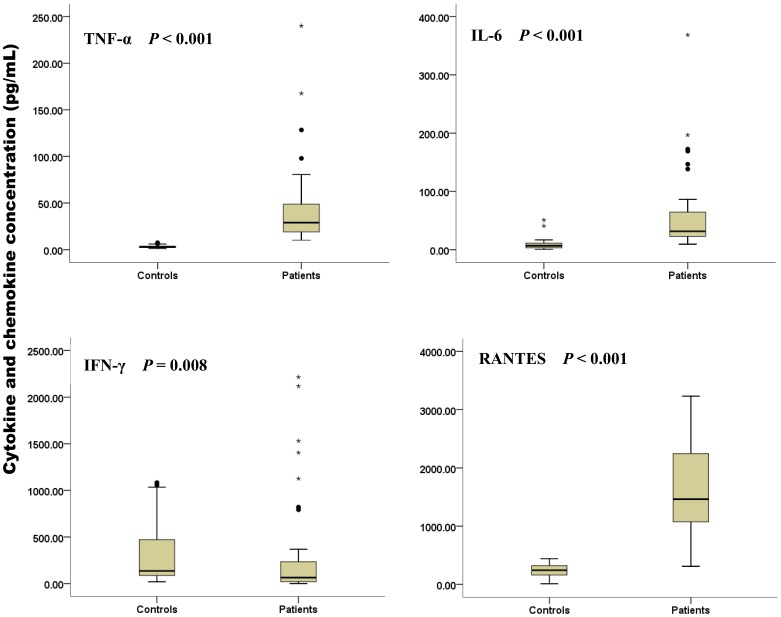
Levels of cytokines (pg/mL) and chemokines (pg/mL) were determined as described and only those with a *P* value of <0.05 are illustrated. Box plots illustrating the significant differences of TNF-α, IL-6, IFN-γ and RANTES in SFTS patients and healthy controls.

**Figure 2:**
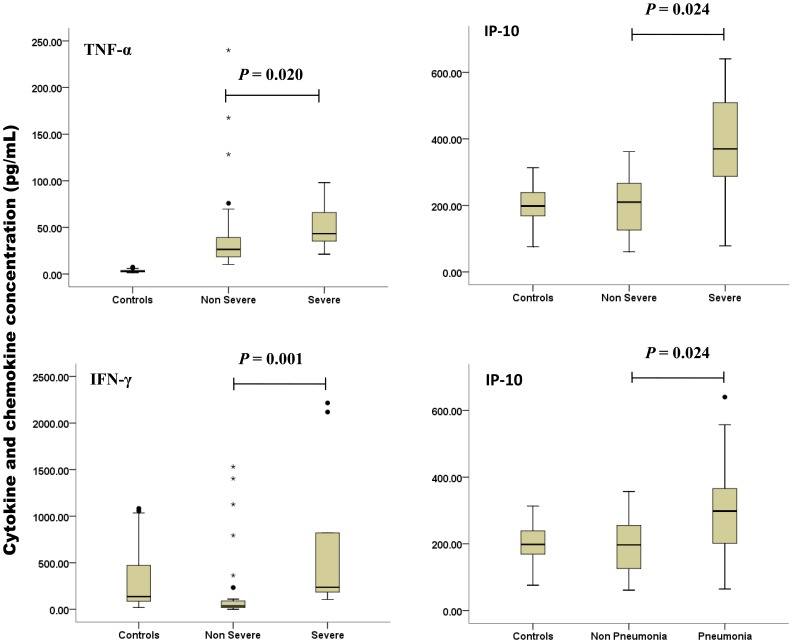
Box plots illustrating the significant differences of TNF-α, IP-10 and IFN-γ in non-severe and severe patients with SFTSV infection and IP-10 in non-pneumonia and pneumonia patients with SFTSV infection.

Serum levels of TNF-α, TGF-β, IL-6, IP-10, IFN-γ and RANTES on admission are shown in Table 3, Figure 1 and Figure 2. Levels of TNF-α, IL-6 and RANTES were significantly higher in patients, and levels of IFN-γ were significantly lower in patients than those in healthy individuals. Levels in the patients with severe and non-severe SFTSV infection were compared. Median TNF-α levels were 43.3 pg/mL (range, 21.3–97.9 pg/mL) and 26.4 pg/mL (range, 10.2–240.1 pg/mL) in the patients with severe and non-severe SFTSV infection, respectively, and the difference was significant (*p* = 0.020). Median IFN-γ levels were 236.4 pg/mL (range, 105.0–2216.1 pg/mL) and 35.4 pg/mL (range, 0.3–1530.8 pg/mL) in the patients with severe and non-severe SFTSV infection, respectively, and the difference was significant (*p* = 0.001). Median IP-10 levels were 369.7 pg/mL (range, 78.3–640.4 pg/mL) and 209.9 pg/mL (range, 60.9–361.5 pg/mL) in the patients with severe and non-severe SFTSV infection, respectively, and the difference was significant (*p* = 0.024). Levels of IP-10 in patients with pneumonia (n = 20) were significantly higher than those in patients without pneumonia (n = 20) (*p* = 0.024). There were no significant differences in IL-6, TGF-β and RANTES levels between patients with severe and non-severe SFTSV infection. Levels of IFN-γ in non-severe cases were significantly lower (*p*<0.001) than values detected in healthy individuals. Although levels of IFN-γ in severe cases were higher than those in healthy individuals, the difference was not significant (median, 236.4 vs 136.0 pg/mL, respectively; *p* = 0.063). Levels of IP-10 in severe cases were significantly higher (*p* = 0.019) than those in healthy individuals (data not shown). Counts of CD4^+^ and CD8^+^ T cells were shown to decrease. The difference in the lymphocyte subgroups was not statistically significant between severe and non-severe cases (data not shown).

**Table 3:** Cytokines and chemokines in severe and non-severe patients infected with SFTSV and healthy individuals.

	Severe cases (n = 9)	Non-severe cases (n = 31)	*P* value	All patients	Healthy individuals	*P* value
	Median (range)	Median (range)		Median (range)	Median (range)	
**TNF-α**	43.3 (21.3–97.9)	26.4 (10.2–240.1)	0.020	29.0 (10.2–240.1)	3.0 (1.4–7.4)	<0.001
**TGF-β**	7.0 (4.2–107.3)	20.7 (3.0–100.1)	0.604	20.6 (3.0–107.3)	25.2 (10.3–63.8)	0.441
**IL-6**	53.9 (23.4–172.5)	31.4 (9.4–368.4)	0.169	31.6 (9.4–368.4)	6.7 (0.5–50.9)	<0.001
**IP-10**	369.7 (78.3–640.4)	209.9 (60.9–361.5)	0.024	225.1 (60.9–640.4)	198.2 (75.8–313.3)	0.055
**IFN-γ**	236.4 (105.0–2216.1)	35.4 (0.3–1530.8)	0.001	64.0 (0.3–2216.1)	136.0 (18.8–1083.0)	0.008
**RANTE**S	1144.8 (419.3–2785.7)	1618.2 (310.7–3231.9)	0.448	1464.0 (310.7–3231.9)	243.7 (11.1–443.1)	<0.001

Data are median, levels in pg/mL.

**Figure 3:**
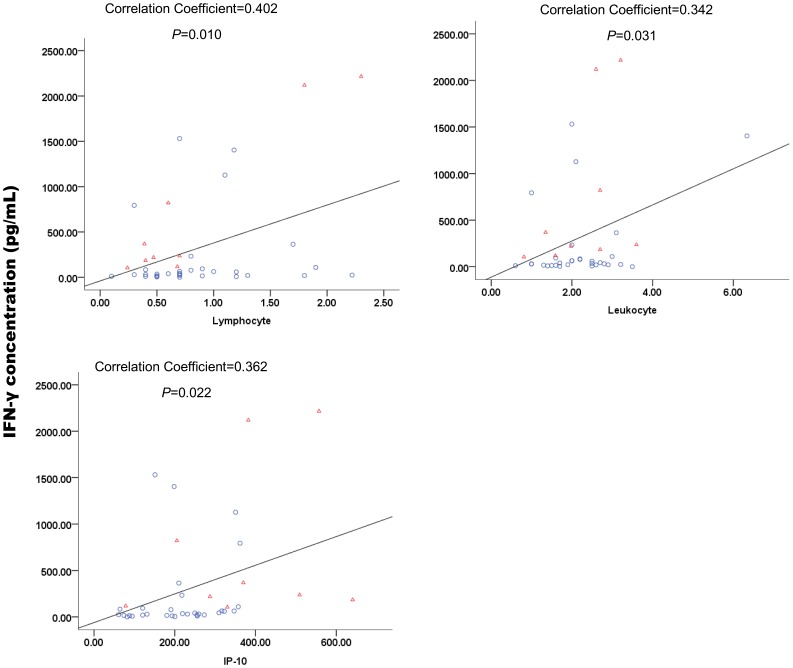
Correlations between variables were assessed using the Pearson test. *P*<0.05 was considered significant. Symbols and colors: red diamonds, severe patients; blue circles, non-severe patients.

Correlation between clinical and laboratory parameters and cytokines and chemokines was analyzed. Only leukocyte and lymphocyte counts positively correlated with the levels of IFN-γ. A positive correlation between IFN-γ and IP-10 levels was observed (Figure 3).

## Discussion

We have presented the cytokine profiles following SFTSV infection in 40 hospitalized patients. The interval between onset and admission was significantly longer in severe cases than in non-severe cases, and may be a risk factor for severity of SFTSV infection. Laboratory findings include higher levels of CRP, LDH and CK, and lower levels of albumin, platelet count and serum calcium in severe cases.

To date, the pathogenesis of SFTSV infection has not been clearly defined. Inflammatory cytokines and chemokines, the first ramification of activation of the innate immune cells, play a role in the pathogenesis of virus infection in animals and humans [6]. It has also been suggested that besides the high levels of viral replication, immunological response might contribute to disease pathogenesis of SFTSV infection [7]. In our prospective study, the most striking phenomenon was the significant increase in plasma TNF-α, IL-6 and RANTES and the significant decrease in plasma IFN-γ in patients with SFTSV infection. The increased levels of TNF-α, IFN-γ and IP-10 are related to the severity of illness. These data, together with the significant correlation found between the levels of IFN-γ and IP-10, suggest that an intense inflammatory response to virus infection, perhaps specifically involving recruitment of inflammatory cells to infected tissues, contributes to disease pathogenesis [8]. Markedly elevated levels of TNF-α and IFN-γ are also correlated with the fatality of Ebola virus infection [9]. TNF-α is also correlated with the severity of hemorrhagic fever with renal syndrome (HFRS) [10], [11]. Pleural and pericardial effusions and hemorrhagic-fever-like symptoms were commonly observed in these patients with SFTSV infection [4]. However, the pathogenesis of vascular leakage is not known. Viral factors can target the endothelium directly or indirectly, and virus-mediated, host-derived soluble factors can cause endothelial activation and dysfunction indirectly [5]. TNF-α produced by monocytes, macrophages and T cells acts on the endothelium, stimulates the production of vasodilating substances, and is an inducer of NO synthase, with important effects on capillary endothelial permeability [11], [12]. IFN-γ and TNF-α have a synergistic effect on endothelial cell cultures *in vitro* by increasing monolayer permeability, which might play a role in capillary leakage [13]. Whether the role of TNF-α in SFTSV infection is the same as that in other viral hemorrhagic fevers is not known.

TNF-α is one of the main proinflammatory proteins, and IL-6 and IFN-γ are also involved in induction of acute inflammatory responses. Levels of cytokines such as IL-6 and IFN-γ in the blood of SFTSV-infected patients are also associated with clinical outcome [4]. In the present study, levels of IFN-γ were shown to increase in all patients, especially in fatal cases. IFN-γ is also a predictive factor for disease severity in dengue patients [14]. Major functions of IFN-γ are activation of macrophages, differentiation of T helper (Th)1 from T cells, inhibition of the Th17 pathway and control of intracellular pathogens [15]. IL-6 plays a major role in host defense mechanisms, including immune responses, acute-phase reactions, and hematopoiesis [16]. In the present study, levels of IL-6 were not associated with severity of illness, but levels of IFN-γ in severe cases were markedly elevated compared with non-severe cases. In contrast, expression of IFN-γ in non-severe cases was suppressed, which suggests that IFN-γ may lead to disease progression. It has also been reported that there is a significant association between the high illness severity phenotype and the IFN-γ^+^874T allele in patients with acute infection with Epstein–Barr virus (EBV), *Coxiella burnetii*, or Ross River virus [17]. The cause of the significantly lower levels of IFN-γ in non-severe cases with SFTSV infection is not yet known.

IP-10, a chemokine synergistically induced in various cell types by type I (IFN-α and IFN-β) and II (IFN-γ) IFNs, lipopolysaccharide, other cytokines and Toll-like receptor ligands, is a chemoattractant for recruiting natural killer and activated T cells into sites of tissue inflammation [18], [19]. The levels of IP-10 in patients were similar to those in healthy individuals, with significantly higher levels in severe cases compared with non-severe cases and healthy individuals. IP-10 is a potent chemoattractant for activated Th1 lymphocytes, and IFN-γ produced during Th1 responses may reflect CD8^+^ T cell activation with production of inflammatory cytokines [20]. Therefore, our findings suggest that SFTSV activates mainly the Th1 immune response against viral invasion. Significantly elevated IP-10 levels were observed in patients with pneumonia, which has also been reported in infection with H5N1 influenza virus and severe acute respiratory syndrome [21], [22]. However, it has been reported that the expression of IP-10 and RANTES induced by hantavirus infection can not increase the permeability in human lung microvascular endothelial cells [23]. IP-10 in patients with H5N1 virus infection might explain the prominent macrophage inflammatory infiltrate in the lungs [24]. In human cutaneous leishmaniasis, IFN-γ production is one of the indicators of a sustained cell-mediated immune response, which is mediated not only through expansion of antigen-specific IFN-γ-producing CD4^+^ Th1 cells, but also through IFN-γ-producing CD8^+^ T cells [25]. Whether IFN-γ produced by CD8^+^ T cell also plays a role in severe patients with SFTSV infection requires further evaluation. The reduced levels of IFN-γ in non-severe patients may be due to the inhibited expression of IFN-γ or production of IFN-γ antagonist during SFTSV infection. Decreased level of IFN-γ in non-severe SFTS patients suggests that expression of IFN-γ during SFTSV infection may reflect the severity of the disease.

Interaction between these cytokines has also been demonstrated. The TNF-α-mediated morbidity or mortality in mouse models of cerebral malaria and bacterial sepsis also can be regulated by IFN-γ [13]. The IFN-γ response is itself regulated by interaction with responses to TNF-α [26]. TNF-α is an early-phase cytokine that acts locally to trigger a cascade of other cytokines including IP-10 and IL-6 [24]. A positive correlation between IFN-γ and IP-10 levels has been observed in these patients.

Association of serum levels of TNF-α and IL-6 with high serum creatinine and low platelet counts in HFRS patients has been reported [11]. Association of serum levels of cytokines was not observed in our study, including high serum CK and creatinine, low platelet and T-cell subgroup (CD4^+^ and CD8^+^ T cells). Upregulated levels of IL-6 are related to increased responses to infection, such as fever, phagocytic cell recruitment, and blood vessel permeability [27]. No correlation between serum cytokines and chemokines levels and body temperature were observed in our study. However, leukocyte and lymphocyte counts were positively correlated with the levels of IFN-γ (Figure 3). Lymphopenia is common in SFTS patients. Apoptosis of lymphocytes can be induced by IFN-γ [28], [29], but levels of IFN-γ were shown to decrease in most patients in this study. How lymphopenia occurs is not clearly understood.

On the basis of our results, we believe that a cytokine-mediated inflammatory response, characterized by cytokine and chemokine production imbalance, plays an important role in the disease progression of patients with SFTSV infection. The levels of Th1 cytokines are correlated with disease severity. Further immunological studies are required to elucidate the role of the immune response in patients’ outcomes.
